# P-Glycoprotein and Androgen Receptor Expression Reveals Independence of Canine Prostate Cancer from Androgen Hormone Stimulation

**DOI:** 10.3390/ijms23031163

**Published:** 2022-01-21

**Authors:** Alexandre Matheus Baesso Cavalca, Andressa Brandi, Ricardo Henrique Fonseca-Alves, Renée Laufer-Amorim, Carlos Eduardo Fonseca-Alves

**Affiliations:** 1 Department of Veterinary Surgery and Anesthesiology, School of Veterinary Medicine and Animal Science, São Paulo State University—UNESP, Botucatu 18618-687, Brazil; a.cavalca@unesp.br (A.M.B.C.); andressabrandi@gmail.com (A.B.); 2 Department of Electrical Engineering, School of Electrical, Mechanical and Computer Engineering, Federal University of Goias—UFG, Goiania 74690-900, Brazil; ricardo_alves@discente.ufg.br; 3 Department of Veterinary Clinic, School of Veterinary Medicine and Animal Science, São Paulo State University—UNESP, Botucatu 18618-687, Brazil; renee.laufer-amorim@unesp.br; 4 Institute of Health Sciences, Paulista University—UNIP, Bauru 17048-290, Brazil

**Keywords:** ABCB1, testosterone, prostatic disease, comparative oncology

## Abstract

Canine prostate cancer (PC) is an aggressive disease, and dogs can be considered comparative models for human PC. In recent years, canine PC has been shown to resemble human castrate-resistant prostate cancer. The influx and efflux of testosterone in prostatic luminal cells are regulated by P-glycoprotein (P-gp). Therefore, human PC generally lacks P-gp expression and maintains the expression of androgen receptors (ARs). However, this co-expression has not previously been investigated in dogs. Therefore, this study aimed to evaluate AR and P-gp co-expression to elucidate these protein patterns in canine prostate samples. We identified AR/P-gp double immunofluorescence co-expression of both proteins in normal luminal cells. However, in canine PC, cells lack AR expression and exhibit increased P-gp expression. These results were confirmed by gene expression analyses. Overall, our results strongly suggest that normal canine prostate testosterone influx may be regulated by P-gp expression, and that during progression to PC, prostatic cells lack AR expression and P-gp overexpress. P-gp expression in canine PC may be related to a phenotype of multiple drug resistance.

## 1. Introduction

The prostate is a hormone-dependent gland with androgens that play a pivotal role in its development and maintenance [1]. Prostate luminal cells express the androgen receptor (AR) in the nucleus, and testosterone acts directly on its receptor, inducing cell proliferation under physiological conditions [2]. The influx and efflux control of steroidal hormones through the plasma membrane has been widely studied in human tissues, and P-glycoprotein (P-gp) plays a central role in this process [3,4,5,6]. P-gp is a transport protein encoded by the multidrug resistance gene MDR1 and belongs to the ATP-binding cassette superfamily [7]. P-gp acts as a pump that induces efflux of xenobiotics and other substances from the cytoplasm to the extracellular space, including the physiological regulation of testosterone in normal luminal prostatic cells [8].

As testosterone levels and AR expression are important for normal prostatic epithelium development and maintenance, it is unsurprising that human prostate cancer (PC) also adopts this mechanism to induce tumor cell proliferation [8]. The central role of the *AR* signaling pathway in PC has been studied for decades, revealing evidence that orchiectomy can induce PC regression. Huggins and Hodges [9] provided a new perspective on this disease’s therapeutic approach. It is well-established that PC expresses AR in the early stages of carcinogenesis, and androgen deprivation is the first-line treatment for human PC [10]. During the early stages of carcinogenesis, PC cells suppress P-gp expression, leading to testosterone accumulation in the cell cytoplasm. Thus, due to AR-positive expression in the nuclei, cytoplasmic testosterone can induce cell proliferation [7]. Therefore, the early stages of human PC express AR and induce P-gp downregulation to maintain high intracellular testosterone levels. However, androgen-resistant tumor cells do not require testosterone for cell proliferation. Thus, these cells increase P-gp expression, leading to a multidrug-resistant phenotype to acquire a more aggressive phenotype [11].

Besides humans, dogs are the only mammals that develop PC with a high frequency, and dogs have been considered a model for human PC study [12]. However, recent studies have indicated that canine PC could be used as a model for castrate-resistant PC (CRPC) [13,14]. The typical human PC expresses AR, NKX3.1, and PTEN, while CRPC generally lacks NKX3.1 and PTEN expression, conferring a more aggressive phenotype. Conversely, several studies have demonstrated that canine PC demonstrates NKX3.1 [14,15,16] and PTEN [13,14,15,17,18,19] downregulation, similar to human CRPC.

Although P-gp is important for the regulation of intracellular testosterone and binding to its receptor in prostatic luminal cells, no previous studies have demonstrated P-gp expression in canine prostate samples or the association of P-gp/AR expression in prostatic tissues. Owing to the importance of P-gp in the plasma membrane in PC development, this study aimed to evaluate AR and P-gp co-expression to elucidate these protein patterns in canine prostate samples. Moreover, we performed an in silico analysis of human PC data regarding AR and P-gp expression, comparing the results with those of the dog’s PC.

## 2. Results

AR expression is pivotal for PC development and maintenance. Testosterone can bind the ARs in the nucleus to stimulate proliferation. This mechanism is physiologically controlled by P-gp, a transmembrane glycoprotein responsible for intracellular testosterone efflux. Therefore, since canine PC is an aggressive disease and generally lacks AR expression, we hypothesize that canine PC will demonstrate low levels of AR and P-gp overexpression. It is phenotypically different from a typical human PC. Thus, because dogs can be considered models of human PC, this model could resemble only human PC with aggressive behavior. In this context, we investigated P-gp and *AR* gene and protein expression in canine tissue samples and performed in silico analysis to investigate P-gp and *AR* gene and protein expression in human PC samples. Moreover, we attempted to associate P-gp and *AR* expression with overall survival and disease-free interval in human PC patients.

### 2.1. AR/P-gp Double Staining in Canine Prostatic Samples

**Figure 1:**
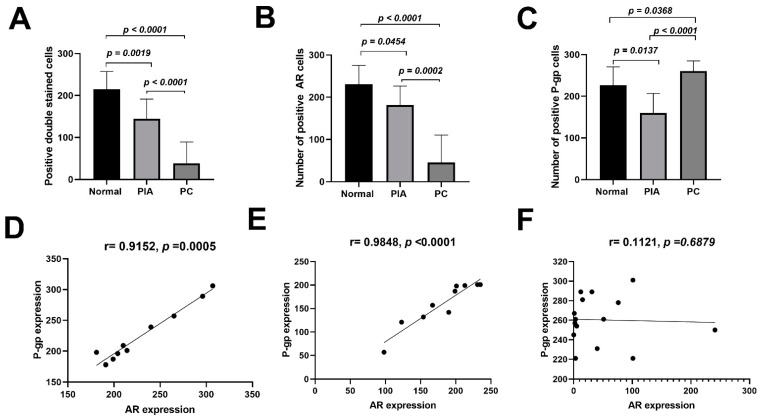
Expression of androgen receptor (AR), P-glycoprotein (P-gp) and doubled stained cells (AR/P-gp) in canine prostatic samples. (**A**): Analysis of variance (ANOVA) revealing high doubled stained cells (AR/P-gp) in normal samples and decreased expression on proliferative inflammatory atrophy (PIA) and prostate cancer (PC) samples. (**B**): High AR expression in normal samples compared to PIA and PC. (**C**): ANOVA analysis revealing higher P-gp expression on PC samples, compared to normal and PIA. (**D**): Positive correlation between the number of AR and P-gp positive stained cells in normal samples, indicating a dependency of both variables. Therefore, in normal prostate, increased AR expression also increases P-gp expression. (**E**): Correlation analysis revealing that samples with higher AR expression also demonstrate higher P-gp expression in PIA samples. Similar to normal samples, in PIA samples, AR and P-gp expression presents dependency, indicating control of P-gp to androgen hormones influx. (**F**): No correlation is found between AR and P-gp expression in PC samples. Thus, in canine PC, no dependency or relation is detected between AR and P-gp expression.

The AR protein was located in the cell nucleus in normal, proliferative inflammatory atrophy (PIA), and PC samples, with cytoplasmic expression in PC samples. P-gp demonstrated membranous expression in normal samples and membranous/cytoplasmic expression in PIA and PC samples. A higher number of AR-positive cells was identified in the normal samples than in the PIA and PC samples (*p* < 0.0001) (Figure 1A). However, P-gp expression was higher in PC samples than in normal and PIA samples (*p* < 0.0001) (Figure 1B). When the number of double-stained cells was evaluated, a higher number of double-stained cells was observed in normal samples compared to PIA and PC samples (*p* < 0.0001) (Figure 1C). In both the normal (Figure 1D) and PIA (Figure 1E) samples, there was a positive correlation between AR and P-gp expression, indicating that samples with higher AR expression also presented higher P-gp expression. Conversely, no correlation between AR and P-gp was detected (r = 0.1121, *p* = 0.6879) in the PC samples (Figure 1F). Individual results regarding AR and P-gp expression in each sample are shown in Supplementary Table S1.

**Figure 2:**
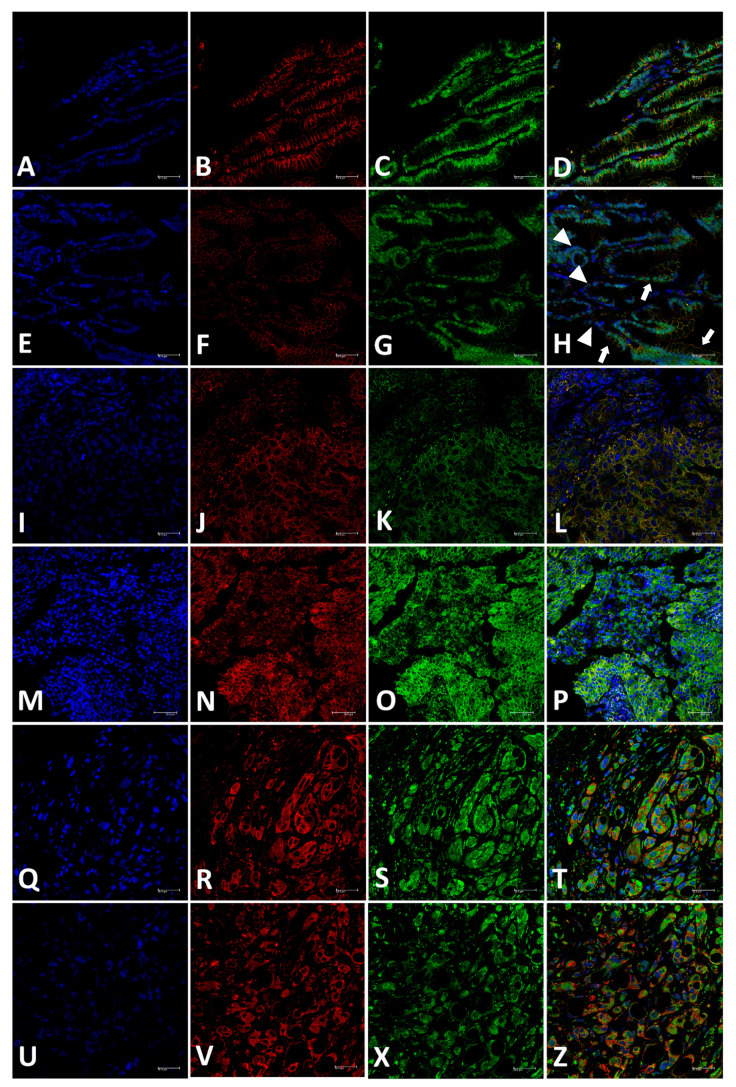
Immunofluorescence analysis of androgen receptor (AR) (green color) and P-glycoprotein (P-gp) (red color) in canine prostatic samples. (**A**–**D**): expression of P-gp (**B**) and AR (**C**) in normal prostate samples and merged image (**D**) demonstrating the doubled stained cells with AR expression in nucleus and P-gp in cell membrane. (**E**–**H**): expression of P-gp (**F**), AR (**G**) and merged image (AR/P-gp) in PIA samples with surrounding normal tissue. The normal prostatic cells are showing positive P-gp expression in the membrane and nuclear AR expression (arrows). Conversely, PIA areas are showing no membranous P-gp and lack of AR expression (nucleus stained with DAPI—blue color) (arrowhead). (**I**–**L**): Canine PC with solid pattern showing cytoplasmic and membranous P-gp expression (**J**) and AR cytoplasmic expression (**K**). In the merged image (AR/P-gp double-stained) (**L**), it is observed lack of nuclear AR (cells stained with DAPI) and cell cytoplasm with yellowish stained (indicating a colocalization of AR and P-gp in cytoplasm). (**M**–**O**) and ‘(**P**): PC samples showing low P-gp expression and high AR cytoplasmic expression with no nuclear expression (**P**–**T**): PC samples showing P-gp membranous expression with AR stained in nucleus and cytoplasm (**T**–**X**), and (**Z**): PC samples revealing membranous P-gp expression with AR cytoplasmic expression (**Z**). Immunofluorescence images, DAPI counterstaining.

Therefore, in the normal canine prostate, both AR/P-gp are expressed, while PC shows increased P-gp expression (including cytoplasmic accumulation) and lacks AR (Figure 2).

### 2.2. AR and MDR1 Molecular Evaluation Indicates Independence of Hormone Regulation

**Figure 3:**
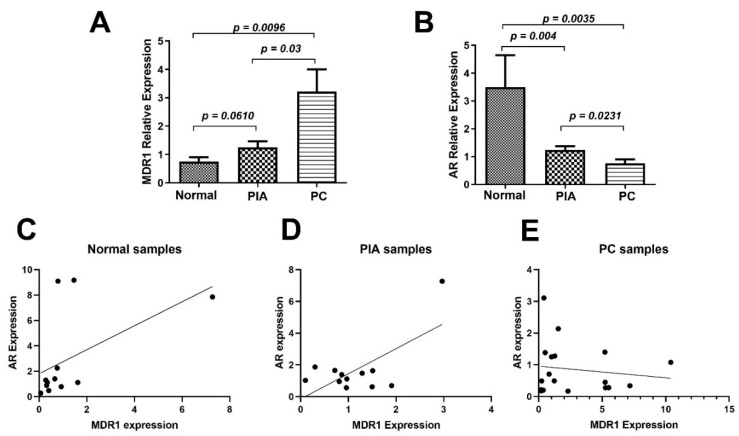
*MDR1* and *AR* gene expression analysis in canine prostatic samples. (**A**): *MDR1* expression revealing higher transcript levels on canine prostate cancer (PC), compared to normal and proliferative inflammatory atrophy (PIA) samples. (**B**): *AR* transcripts in canine prostatic samples. A higher *AR* expression on normal samples, compared to normal and PIA samples is observed. (**C**): Linear regression analysis revealing a positive association between *MDR1* and *AR* transcripts. (**D**): Canine PC lacking association between *MDR1* and *AR* transcripts. (**E**): Linear regression revealing positive association between *MDR1* and *AR* transcripts.

*MDR1* and *AR* gene expression was assessed in normal, PIA, and PC samples, and revealed a higher *MDR1* expression in PC than in normal and PIA samples (*p* = 0.0187) (Figure 3). However, *AR* transcripts were higher in normal samples than that in PIA and PC samples. We also investigated the correlation between *MDR1* and *AR* transcripts in the normal, PIA, and PC samples. In normal samples, linear regression analysis revealed a positive linear regression between *MDR1* and *AR* transcripts (r^2^ = 0.5245) (Figure 3). In PIA samples, a linear positive regression association was also found between MDR1 and AR transcripts (r^2^ = 0.4477). In canine PC, no correlation or linear regression association was observed. These results indicate that canine PC lacks testosterone stimulus (lacking nuclear AR) and overexpresses *MDR-1* transcripts (leading to high P-gp expression), demonstrating its independence in hormone regulation.

In human PC, *MDR1* hypermethylation is a well-known mechanism for decreasing P-gp expression, leading to testosterone influx. However, no previous studies have investigated *MDR1* hypermethylation in canine PC. Therefore, we performed pyrosequencing of normal, PIA, and PC samples to investigate *MDR1* methylation status. No methylation of the MDR1 promoter region was observed in normal samples, indicating that methylation is not a physiological regulatory mechanism. In PIA samples, the methylation percentage was 3.8% and no methylation was detected in *MDR1* in PC samples.

### 2.3. AR and P-gp Expression in Human PC

**Figure 4:**
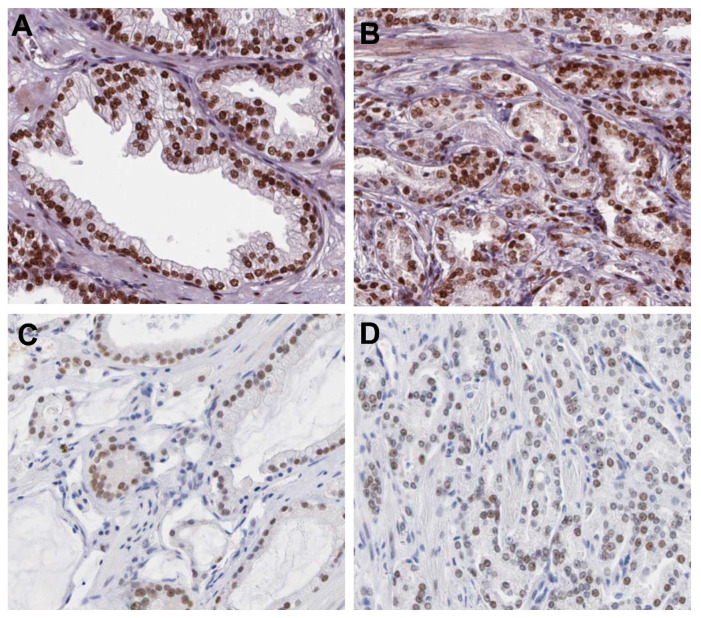
AR immunohistochemical expression human prostatic samples. (**A**): Normal prostate gland demonstrating strong nuclear AR expression in luminal cells. (**B**): A high-grade prostate cancer (PC) showing strong nuclear AR expression. (**C**): A low grade PC sample showing moderate AR nuclear expression. (**D**): A high-grade PC showing moderate nuclear AR expression. Image credit of the IHC images: Human Protein Atlas, www.proteinatlas.org. Image available at the following URL: https://www.proteinatlas.org/ENSG00000169083-AR/pathology/prostate+cancer, accessed on 16 November 2021.

**Figure 5:**
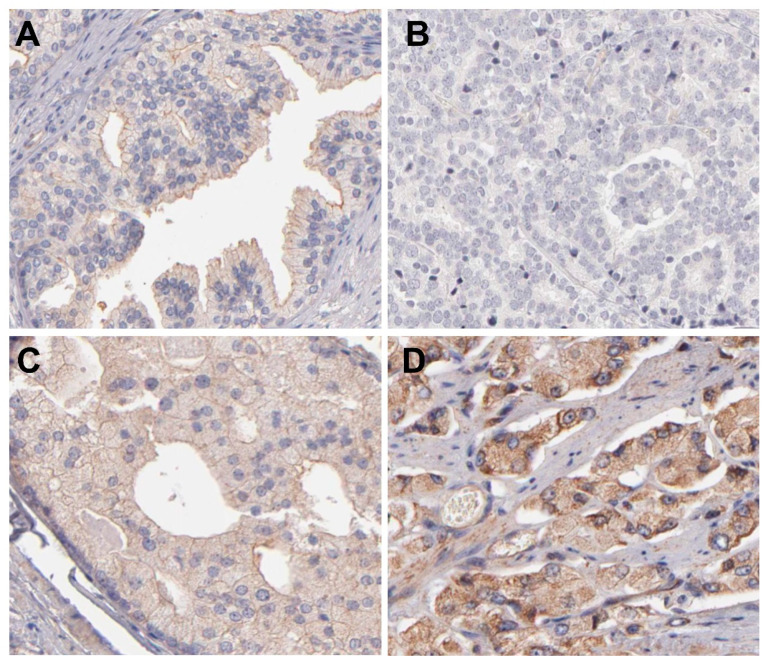
P-glycoprotein (P-gp) immunohistochemical expression human prostatic samples. (**A**): Normal prostate gland showing weak membranous P-gp expression by luminal cells. (**B**): A high-grade prostate cancer (PC) showing no P-gp expression. (**C**): A PC representing moderate cytoplasmic and membranous P-gp expression. (**D**): A high-grade PC showing strong membranous and cytoplasmic P-gp expression. Image credit of the IHC images: Human Protein Atlas, www.proteinatlas.org. Image available at the following URL: https://www.proteinatlas.org/ENSG00000085563-ABCB1/pathology/prostate+cancer#img, accessed on 16 November 2021.

To provide a more reliable comparison between canine and human PC, we performed in silico analysis of AR and P-gp in human PC to provide a more reliable comparison between canine and human PC. First, we investigated AR and P-gp expression patterns in normal prostate and PC samples using the Human Protein Atlas. In the normal human prostate, AR demonstrates strong nuclear expression, and this expression pattern remains in PC, which shows moderate to strong AR expression (Figure 4). Conversely, P-gp showed weak membranous/cytoplasmic expression in normal prostatic samples, while PC showed negative to moderate expression (Figure 5).

**Figure 6:**
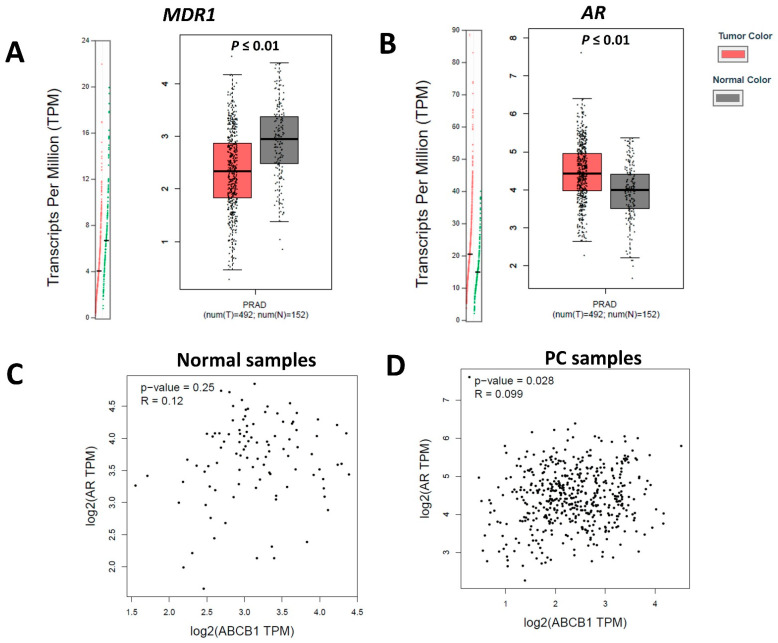
*MDR1* and *AR* transcriptional evaluation on human prostate samples. (**A**): Higher MDR1 gene expression in normal prostatic samples compared to prostate cancer (PC) samples. The normal samples demonstrate higher transcripts per million (TPM) (green dots) than PC (red dots). (**B**): *AR* gene expression in normal prostate and PC cases. Higher AR expression on PC samples is observed. The normal samples demonstrate lower TPM (green dots) than PC (red dots). (**C**): Correlation analysis between *MDR1* and *AR* gene expression in normal prostatic samples showing no association. (**D**): Positive low correlation between *MDR1* and *AR* transcripts on human PC samples. The gene expression dotplot is generated using the GEPIA database (http://gepia.cancer-pku.cn/about.html, accessed on 16 November 2021). PRAD: PC dataset.

*MDR1* and *AR* gene expressions were also assessed in human normal prostate tissue (*n* = 152) and compared to PC (*n* = 492). *MDR1* expression was lower in human PC samples than that in normal samples (Figure 6). However, *AR* transcripts were higher in the PC samples than in the normal samples (Figure 6). This indicates an *MDR1*-low/*AR*-high profile in human PC. Correlation analysis was performed between MDR1 and AR transcripts in normal human prostate samples and PC. There was no correlation between *MDR1* and *AR* transcripts in normal prostatic samples (r = 0.12, *p* = 0.25), but there was a positive correlation between *MDR1* and *AR* transcripts in human PC samples (r = 0.099 and *p* = 0.028) (Figure 6).

Regarding survival analysis, no association was found between patients with low or high AR expression and *MDR1* expression with overall survival and disease-free interval.

## 3. Discussion

For many years, canine PC has been considered a model for human PC, and several studies have proposed and evaluated the importance of the canine model from a single health perspective [20,21,22,23,24,25]. However, based on our previous results [13,14], we propose that dogs can be used in comparative studies considering the particularities of PC in both species. In human PC, tumors generally express ARs and respond to androgen deprivation therapy [2]. Only one set of tumors became resistant to androgen deprivation and required additional aggressive treatment [26]. Nevertheless, canine PC appears to originate under low testosterone stimulation and negative to low AR expression [13,19,27]. Therefore, dogs could not be a model for the usual human type of PC (luminal phenotype with AR-positive expression). Thus, we investigated the role of P-gp and AR receptors in canine prostatic samples to comprehend the role of these markers in the canine prostate.

As AR plays a pivotal role in normal prostate cells and PC cells [2], several studies have investigated the role of *MDR1* and its protein (P-gp) in human PC. It was previously hypothesized and demonstrated that the MDR1 promoter region is hypermethylated during PC development, leading to a higher influx of testosterone in prostatic neoplastic cells [28,29,30,31]. Based on this information, we performed an in silico analysis of previous literature to confirm our hypothesis. The phenotype of the human normal prostate shows low P-gp protein expression and strong AR expression in the nucleus of luminal cells. Moreover, canine normal prostate cells also demonstrated nuclear AR expression; however, different from human normal prostate cells showed higher membranous P-gp expression. Since P-gp can bind testosterone, its overexpression is important for prostate cell regulation. Interestingly, in canine PIA, loss of AR expression and loss of membranous P-gp (cytoplasmic accumulation in this lesion) were observed.

Canine PIA was previously associated with decreased gene and protein AR expression and mutations, leading to AR downregulation [23]. Our doubling staining results reinforced this previous finding and demonstrated that PIA progression could lead to PC development from cancer cells independent of testosterone. Usual-type human PC appears to progress from high-grade prostatic intraepithelial neoplasia (HGPIN), which is a controversial lesion. Some authors believe that PIA can progress to PC or HGPIN, being a PC precursor lesion [32,33]. However, some researchers have not demonstrated this association [34,35]. In canine prostatic pathology, HGPIN was previously reported to be frequently associated with canine PC [36,37]. However, HGPIN appears to have a very low frequency in dogs [12]. Godoy et al. [23] investigated 469 prostate tissues from different dogs and found only 14 tissue samples with PIN. Regarding PIA, the authors found 171 dogs with these lesions and 84 dogs with PC. Among the dogs with PC, 51 of 84 had concomitant PIA.

In addition to decreased AR protein expression, canine PC also showed decreased *AR* transcript levels. However, we did not obtain serum samples from these dogs to investigate testosterone levels. Since the tumors showed AR gene and protein downregulation, we believe that these tumors are independent of androgen hormones. In human PC, even with tumor androgen resistance, cancer cells generally express AR. This is a particular difference between human and canine PCs. Even with different phenotypes, tumors do not respond to androgen deprivation in both cases. Therefore, we strongly support the use of dogs as a model for this specific set of human tumors.

Canine PC is considered an aggressive disease with a low response to systemic therapies [12]. One reason for this phenomenon may be P-gp overexpression. *MDR1* overexpression is widely known as a resistance phenotype in different tumor subtypes [38,39]. Thus, our results indicated the importance of P-gp in canine PC resistance. Thiemeyer et al. [40] performed a molecular characterization of canine PC, revealing MDR1 overexpression (log_2_ fold change 3.6) and AR downregulation (log_2_ Fold Change −3.1), supporting the idea that canine PC develops under low androgen hormone stimulation and P-gp overexpression. Moreover, this phenotype may be related to multidrug-resistant tumors. Interestingly, Azakami et al. [41] investigated the growth of a canine PC cell line (CHP-1) under dihydrotestosterone stimulation, demonstrating low AR expression in this cell line and reinforcing its growth independent of androgen hormone stimulation.

From the clinical perspective of dogs with PC, our results also provide new information regarding tumor resistance. Although P-gp is responsible for androgen hormone efflux in the prostate, it is also associated with multiple drug resistance. Canine PC is a highly aggressive disease, with aggressive clinical behavior and a lack of response to chemotherapy [42,43]. P-gp overexpression may explain the lack of response to standard chemotherapy. To the best of our knowledge, this is the first study to report P-gp overexpression in canine PC. Therefore, future studies associating P-gp expression with standard chemotherapy responses could help to understand the mechanism involved in this phenomenon.

Another important aspect associated with canine PC’s clinical behavior is the cytoplasmic expression of AR. Non-nuclear AR expression was recently reported in canine PC [19,44]. However, in human cancers, including PC, non-nuclear AR signaling has been associated with aggressive characteristics, including epithelial-mesenchymal transition and metastasis [45,46]. AR nuclear expression is associated with hormone-dependent signaling pathways; however, AR splice variants can be found with different cellular localization, with a clinical impact on human patients [47]. Thus, as occurs in some human cancers, including PC, AR cytoplasmic expression can be associated with different signaling pathways, leading to aggressive tumor behavior.

Therefore, our results strongly suggest that canine PC is generally independent of androgen hormone stimulation and that dogs may be an interesting model for this set of tumors in humans.

## 4. Material and Methods

### 4.1. Ethical Approval

This study was performed in accordance with national and international recommendations for the care and use of animals [48]. All procedures were performed after receiving approval from the Institutional Ethics Committee on Animal Use (CEUA, # 107/2015), and all owners signed a written consent form, allowing the sample to be used in the research.

### 4.2. Tumor Samples

This study included 35 prostate samples from the veterinary pathology archive. Only samples from intact dogs with paraffin-embedded tissue for immunohistochemistry and paired frozen samples for molecular analysis were included in this study. Ten normal prostate, 10 proliferative inflammatory atrophies, and 15 prostatic carcinoma samples were selected. For each diagnosis, hematoxylin and eosin staining was performed, and carcinomas were classified according to Palmieri et al. [12]. The PIA classification was based on Godoy et al. [23].

### 4.3. Double Immunofluorescence

Double immunofluorescence was performed based on a previously reported technique for double immunohistochemistry [49] and immunofluorescence [50] with modifications. Briefly, a two-color immunofluorescence stain was performed using P-glycoprotein (green color) and AR (red color) antibodies. Tissue samples were deparaffinized, and antigen retrieval was performed using citrate buffer solution (pH 6.) in a commercial pressure chamber (Dako, Carpinteria, CA, USA) for 30 s at 125 °C, followed by 25 min at 94 °C, and cooling for 15 min. Then, unpacific binding proteins were blocked using a commercial solution for 30 min (Dako, Carpinteria, CA, USA). Anti-rabbit polyclonal AR (1:50) was purchased from Santa Cruz Biotechnology (Santa Cruz, CA, USA). Alexa Fluor 484 (Invitrogen, Carlsbad, CA, USA) at a 2 μg/mL dilution in phosphate-buffered saline (PBS) for 1 h and the slides were washed using PBS solution. The second primary antibody was applied (1:700 monoclonal mouse anti-P-gp clone C494, BioLegend, San Diego, CA, USA) overnight. Then, the secondary goat anti-mouse antibody was conjugated with Alexa Fluor^®^ 594 (BioLegend, San Diego, CA, USA), for one hour. Slides were counterstained with 4-6-diamidino-2-phenylindole (DAPI; Sigma, Portland, OR, USA) and evaluated using a laser scanning confocal microscope (Leica Biosystems, Wetzlar, Germany).

### 4.4. Gene Expression

Tissue macrodissection, mRNA extraction, cDNA synthesis, and qPCR were performed as in previous literature [19]. Briefly, mRNA was extracted using the RecoverAll™ Total Nucleic Acid Kit (Ambion, Life Technologies, MA, USA), and mRNA quantity was evaluated using a spectrophotometer (NanoDrop ND-1000, Thermo Fisher, Waltham, MA, USA). The primer sequences for MDR1 were as follows: 5′CGCAACCTCCACCGAGAA3 (forward) and 5′CGCAACCTCCACCGAGAA 3′ (reverse). The sequences of endogenous HPRT and AR genes have been previously published [19]. qPCR for MDR1, AR and HPRT (endogenous) genes was conducted in a total volume of 10 μL containing Power SYBR Green PCR Master Mix (Applied Biosystems; Foster City, CA, USA), 1 μL of cDNA (1:10), and 0.3 μM of each primer. The reactions were performed in triplicate in 384-well plates using QuantStudio 12 K Flex Thermal Cycler equipment (Applied Biosystems, Foster City, CA, USA). The amplification reaction conditions for all primers were 40 cycles of 15 s at 94 °C and 1 min at 60 °C. PCR product specificity was determined using the dissociation curve for all experiments. The relative gene expression was quantified using the 2^−ΔΔCT^ method.

### 4.5. Quantitative Bisulfite Pyrosequencing

Pyrosequencing analysis was performed to evaluate the frequency of MDR1 promoter methylation according to a previous study [51]. Each diagnosis was confirmed, and pyrosequencing was performed as previously described [43]. Briefly, bisulfite conversion of genomic DNA was performed using an EZ DNA Methylation-Gold Kit (Zymo Research Corporation, Irvine, CA, USA). The forward (5′ GGTTTGGGTTTTTTGGAGT 3′) and reverse (5′ CCTCCTAAAACTCCAACCT 3′) primers of MDR1 CpG island (Gene ID: 403879) were amplified by PCR (HotStarTaq Master Mix kit, Qiagen) and pyrosequencing was performed using a sequencing primer (5′ TATATTTTGGTGTTTTTG 3′) following the manufacturer’s instructions (PyroMark ID Q96, Qiagen and Biotage, Uppsala, Sweden).

### 4.6. P-Glycoprotein and AR Expression in Human PC

As dogs are considered an interesting model for human PC, we performed an in silico analysis of P-gp and AR gene and protein expression in human PC. First, we evaluated P-gp and AR expression protein patterns using the Human Protein Atlas (THPA) database (https://www.proteinatlas.org/, accessed on 10 November 2021) [52]. In this analysis, protein localization was evaluated, and the protein pattern was divided into the following categories: negative, low, moderate, or high. In this step, 43 images of P-gp immunoexpression from PC-affected patients and five normal prostate samples were evaluated. For AR immunoexpression, 30 PC tissue and three normal prostate images were available for analysis. We then analyzed the *MDR1* and *AR* gene expression PC samples using The Cancer Genome Atlas (*n* = 492) samples compared to normal prostate samples from the Genotype-Tissue Expression project (https://gtexportal.org/home/, accessed on 10 November 2021) matched (*n* = 152), using the Gene Expression Profiling Interactive Analysis (GEPIA) (http://gepia.cancer-pku.cn/, accessed on 10 November 2021) [53]. The criteria used for this analysis were a *p*-value cutoff of 0.01, Log_2_FC cutoff of 1, and jitter size of 0.4 with data presented on the log scale. Survival analysis was performed using a dataset containing 492 PC (PRAD) samples available from both the GEPIA and THPA portals. Using GEPIA, survival analysis was performed using MDR1 and AR median expression as cut-offs to classify patients as high or low expression. Pearson’s correlation between MDR1 and AR transcripts was also determined using GEPIA. The confidence interval was set to 95%, and axis units were provided in months. The disease-free interval was calculated using the same parameters.

### 4.7. Statistical Analysis

Immunofluorescence slides were analyzed by counting the total number of cells (based on DAPI counterstaining) and evaluating the number of P-gp-positive cells, AR-positive cells, and P-gp/AR double-stained cells separately. The Mann–Whitney test or analysis of variance (ANOVA) was applied to identify statistical differences. For gene expression, the Mann–Whitney test and ANOVA were used to investigate statistical differences. Linear regression analysis and Spearman’s correlation tests were used for comparisons between two variables. Statistical analyses were performed using GraphPad Prism 5 v.5.0 (GraphPad Software Inc., La Jolla, CA, USA). The Kruskal–Wallis or Mann–Whitney U test was used to compare MDR1 and AR transcript levels between the normal and PC samples. Statistical significance was set at *p* < 0.05. The correlation coefficients (r) were interpreted according to Pett, Lackey and Sullivan [54], dividing the classifications into weak (0 < r > 0.29), low (0.30 < r > 0.49), moderate (0.49 < r > 0.60), strong (0.61 < r > 0.89) or very strong (0.90 < r > 1); whether they were positive or negative.

## 5. Conclusions

Overall, our results strongly suggest that normal canine prostate testosterone influx may be regulated by P-gp expression, and during progression to PC, prostatic cells lack AR expression and overexpress P-gp. P-gp expression in canine PC may be related to the phenotype of multiple drug resistance.

## Data Availability

Supporting data are available in this manuscript text or the supplementary data.

## Supplementary Materials

The following are available online at https://www.mdpi.com/article/10.3390/ijms23031163/s1.
